# At the human-forest interface

**DOI:** 10.1038/s41467-018-03586-1

**Published:** 2018-03-21

## Abstract

We have a long-shared history with forests and the riches they provide, but their capacity to give is not endless and their future is under increasing threat. On the International Day of Forests, we consider our interactions with them, and how science may guide us towards living sustainably with these vital and iconic ecosystems.

The histories of humans and forests have long been entwined. For millennia, forests have held an allure as places of spirituality and striking imagery. They have inspired human traditions and folklore, appearing as symbols of knowledge, fertility, and life. However, the value of the forest extends far beyond the cultural and the aesthetic; they bridge many domains that are crucial to environmental health and human wellbeing.

**Figure:**
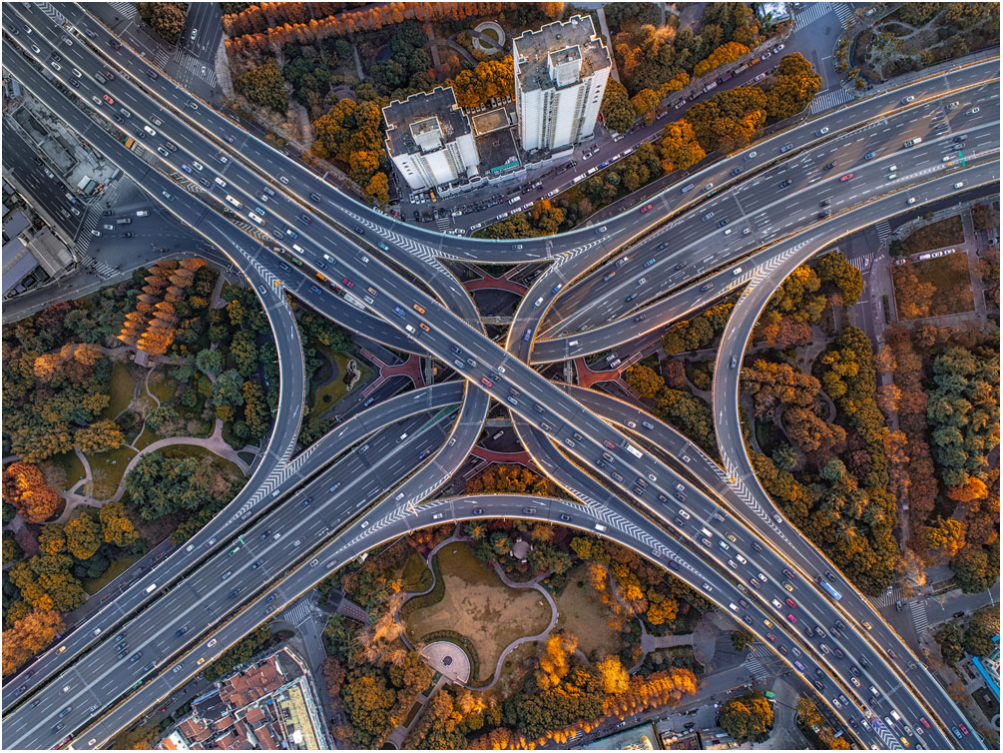
DANNY HU/Moment/Getty

“With an estimated 1.6 billion people depending on forests for their livelihoods, the protection of forests and human well-being is a delicate balancing act with no easy solutions.”

We have long appreciated forests as sources of food, fuel, materials, and land, but advances in our scientific understanding have revealed the crucial, less visible services they also offer. They serve as a giant carbon sink, removing carbon dioxide from the atmosphere and locking it away in their woody biomass. Their vast subterranean networks of roots bind soils, prevent erosion, and grant protection against flooding and landslides. They influence local weather patterns, lowering scorching temperatures in the tropics and driving local cloud systems that provide life-giving rainfall. In short, rich in tangible resources and intangible services, forests are vital to the health of the planet.

As the centuries passed, our appetite for their assets became unsustainable. Since the start of civilisation, intensified logging, the advance of agricultural fields, and urban sprawl have seen 46% of the world’s forests cleared^1^. Forest goods now support a multibillion-dollar industry, upon which millions of people depend. However, despite the commendable efforts of international agencies and national governments, the sustainable management of these valuable resources remains a challenge, with 7.6 million hectares of forest still lost annually, primarily from the tropics^2^.

Safeguarding the environmental services forests afford while continuing to benefit from their many riches in a sustainable fashion has become a “wicked problem”^3^. Forests are interconnected systems, with complex interdependencies: afforestation schemes that attempt to replace lost ecosystems can result in excessive water demands and shortfalls for society^4^; small-scale, selective logging can fragment forests and expose edges that bleed carbon and biodiversity^5,6^; banning products that drive deforestation, such as palm oil, can negatively impact the millions who depend on the industry. Forest systems transcend disciplinary boundaries and their successful management will require cross-discipline collaboration and a holistic understanding of their complexity^7^.

Agroforestry is one approach that seeks to manage forest services and agriculture together. An ecologically based natural resource management system, agroforestry integrates environmental, and socio-economic principles in an effort to cultivate trees and shrubs alongside crops. The trees reduce soil erosion, improve fertility, and increase water availability, making them better suited for crops and can protect against, or even reverse, land degradation^8^. Whilst such projects will not wholly replicate the benefits of the original forests, they can be a compromise in regions where the livelihoods of local landowners depend on productive land.

Socio-economic disciplines have also helped devise policies to protect existing forests while supporting local economies, such as payments for ecosystem service (PES). These schemes offer financial incentives for landowners to protect and manage their forests, allowing wider society to benefit from their water management, carbon capture, and biodiversity. It is difficult to assess the effectiveness of PES schemes, though reduced tree cover loss has been observed in Mexico following the implementation of its PES program^9^. In addition, the grounding of invisible benefits offered by forests in more practical and economic terms may promote the value of their services, making forests a more attractive feature for local landowners^10^. Many questions regarding the success of such projects remain, and continuing research seeks to improve these schemes to benefit both humanity and forest ecosystems.

However, increasing numbers of us no longer live alongside forests. The allure of cities is expected to see their populations increase from 3.5 to 6 billion by 2050. As cities become increasingly congested, water-scarce, flood-prone, and polluted, they are under ever-growing strain to provide a good quality of life, while minimising environmental impact, a challenge that the UN’s Sustainable Development Goals seek to address. Here, trees have a role to play. International Day of Forests 2018 (http://www.fao.org/international-day-of-forests/en/) champions ‘forests and sustainable cities’, an accelerating movement that seeks to enrich urban environments with green spaces. Many cities have embraced urban forests, lacing their streets and parks with trees, their buildings hosting gardens on their rooftops and, increasingly, along their vertical surfaces. These are valued for offering a reprieve from urban living, offering places to gather and enjoy healthy pursuits, buffering city noises, and as sources of both food and fuelwood. But like their rural counterparts, urban forests bring a myriad of invisible benefits. By stripping the air of both pollution and atmospheric carbon, they help improve air quality and counter carbon emissions. Their canopies help to buffer temperature extremes, reducing the demand for energy to heat and cool our homes. Immense root systems slow the deluge of rainwater rushing straight into rivers, protecting against floods and reducing costs of stormwater processing^11^. As our appreciation for these services grow, many cities are turning to forests to help solve the sustainability problem. Urban forest projects are a growing feature of cities worldwide, but sustainability cannot be acquired from the simple planting of trees. In order to meet this challenge, urban projects must assemble a broad array of knowledge and expertise, and must possess clear objectives, integrating forestry into city planning programs.

In an increasingly urbanising world and with all the challenges that this brings, we cannot treat humans and forests in isolation. By revisiting the value of forests, we come closer to striking a balance and achieving a sustainable way of living. There is hope yet that these grand sentinels will endure and continue to inspire future generations.
